# The importance of organizational characteristics for improving outcomes in patients with chronic disease: a systematic review of congestive heart failure

**DOI:** 10.1186/1748-5908-5-66

**Published:** 2010-08-25

**Authors:** Luci K Leykum, Michael Parchman, Jacqueline Pugh, Valerie Lawrence, Polly H Noël, Reuben R McDaniel

**Affiliations:** 1 South Texas Veterans Health Care System and Department of Medicine, University of Texas Health Science Center at San Antonio, San Antonio TX, 78229, USA; 2 South Texas Veterans Health Care System and Department of Family and Community Medicine, University of Texas Health Science Center at San Antonio, San Antonio TX, 78229, USA; 3 McComb's School of Business, University of Texas at Austin, Austin TX, USA

## Abstract

**Background:**

Despite applications of models of care and organizational or system-level interventions to improve patient outcomes for chronic disease, consistent improvements have not been achieved. This may reflect a mismatch between the interventions and the nature of the settings in which they are attempted. The application of complex adaptive systems (CAS) framework to understand clinical systems and inform efforts to improve them may lead to more successful interventions. We performed a systematic review of interventions to improve outcomes of patients with congestive heart failure (CHF) to examine whether interventions consistent with CAS are more likely to be effective. We then examine differences between interventions that are most effective for improving outcomes for patients with CHF versus previously published data for type 2 diabetes to explore the potential impact of the nature of the disease on the types of interventions that are more likely to be effective.

**Methods:**

We conducted a systematic review of the literature between 1998 and 2008 of organizational interventions to improve care of patients with CHF. Two independent reviewers independently assessed studies that met inclusion criteria to determine whether each reported intervention reflected one or more CAS characteristics. The effectiveness of interventions was rated as either 0 (no effect), 0.5 (mixed effect), or 1.0 (effective) based on the type, number, and significance of reported outcomes. Fisher's exact test was used to examine the association between CAS characteristics and intervention effectiveness. Specific CAS characteristics associated with intervention effectiveness for CHF were contrasted with previously published data for type 2 diabetes.

**Results and discussion:**

Forty-four studies describing 46 interventions met eligibility criteria. All interventions utilized at least one CAS characteristic, and 85% were either 'mixed effect' or 'effective' in terms of outcomes. The number of CAS characteristics present in each intervention was associated with effectiveness (p < 0.001), supporting the idea that interventions consistent with CAS are more likely to be effective. The individual CAS characteristics associated with CHF intervention effectiveness were learning, self-organization, and co-evolution, a finding different from our previously published analysis of interventions for diabetes. We suggest this difference may be related to the degree of uncertainty involved in caring for patients with diabetes versus CHF.

**Conclusion:**

These results suggest that for interventions to be effective, they must be consistent with the CAS nature of clinical systems. The difference in specific CAS characteristics associated with intervention effectiveness for CHF and diabetes suggests that interventions must also take into account attributes of the disease.

## Background

Successful management of chronic disease in routine practice is an elusive task [1,2]. As the number of patients with chronic medical illness and the literature regarding their optimal management have grown, attempts have been made to improve their care by implementing new models of care delivery. Implementation of these models involves intervening in specific ways in clinical settings and organizations, and each model has organizational elements that are considered necessary for model implementation. For example, in the chronic care model, delivery system design and information systems are among the elements that are specifically identified [3].

The number of interventions on an organizational level to improve delivery of care and outcomes for patients with chronic disease has grown. However, these care models and organizational strategies have not met with uniform success [4-14]. We believe that an important reason for this variation in outcomes is that interventions do not adequately take into account the characteristics of clinical systems in which patients receive care.

Clinical microsystems are the building blocks of healthcare delivery: the individual clinics, units, or other areas where care is delivered. The complex adaptive system (CAS) framework has been applied to clinical microsystems as a theoretical model for better understanding them [15-22]. This framework suggests that clinical settings are environments in which individuals learn, inter-relate, self-organize, and co-evolve in response to changes in their internal and external environments, in turn shaping those environments [15,19]. Because inputs and outputs in CAS may not be proportional or predictable, interventions that are successful in one setting may not be successful in another. However, evidence suggests that interventions congruent with the CAS framework and characteristics are in general more likely to be effective [21-25].

The insight that clinical settings are CASs is important to the field of implementation research, as it provides guidance for how to approach disseminating research findings into routine care. The CAS framework suggests that local contexts and local interactions between individuals are critical considerations in designing interventions, and that leveraging these may lead to improvements in system performance. However, we wanted to expand on this insight by exploring the possibility that interventions must also be congruent with the nature of the disease or diseases of the patients being cared for. Diseases may mediate the way that interventions influence a patient's care. The level of complexity of different diseases, and the ways that chronic diseases impact patients' lives, varies greatly depending on the type of disease, leading to the need for different approaches. For example, self-monitoring in diabetes may be more difficult than congestive heart failure (CHF) because it may be more difficult to monitor carbohydrates and calories than salt, and involves the pain of fingersticks versus standing on a scale to check weight. Therefore, the most effective patterns of communication between patients with type 2 diabetes and their providers may be different from those for patients with CHF, which in turn may affect the way that providers and staff interact and structure the delivery of care for those groups of patients. Because of this, we believe that for interventions whose goal is to improve the performance of a clinical system to be most effective, they must take into account not only the nature of the system, but also the nature of the disease.

The purpose of this paper is twofold. First, we build on the literature suggesting that interventions consistent with CAS are more likely to be effective [22] by conducting a systematic review of organizational interventions focused on improving care of patients with CHF. This work builds on our previously published systematic review of interventions to improve outcomes for patients with Type 2 diabetes, expanding the data regarding the importance of considering health care settings as CAS beyond a single chronic disease. Like diabetes, CHF is a common disease whose management is broadly relevant. We also chose CHF because of the growing number of studies of interventions to improve CHF outcomes through changing the way that care is delivered in clinical settings.

Our second purpose is to compare the findings of the specific types of interventions that appear to be most effective for CHF and diabetes, to identify differences in the specific CAS characteristics associated with more effective outcomes for each disease. We hypothesized that there are fundamental differences between CHF and diabetes in terms of their impact on patients' day-to-day lives, the behaviors that are required for their successful management, and the structure of care delivery that best supports successful management.

## Methods

### Systematic review of organizational interventions for CHF

Our methods mirrored those in our previously published systematic review of organizational interventions to improve care of patients with type 2 diabetes [22]. Specific elements are as follows.

### Search strategy

We defined organizational interventions as those that explicitly attempt to affect or change organizational structures or processes to implement evidence-based practice. Our search strategy was based on four components: the strategy developed by the Effective Practice and Organization of Care (EPOC) Group of the Cochrane Collaboration [26]; additional search terms for types of organizational interventions not included in EPOC, such as total quality improvement, PDSA (Plan-Do-Study-Act), and practice redesign; additional search terms identified in recent systematic reviews of quality improvement initiatives; and bibliographies and Medline indexing terms of relevant publications.

To focus the search on CHF, we added disease-specific MeSH and text word terms, ran a preliminary search, and reviewed 2,559 titles and abstracts (determined by saturation, until no further new terms were identified), for additional text word terms. The search terms are listed in Additional File 1. We did not search the management literature, nor did we seek out unpublished data. We searched Medline from 1989 through 17 July 2008.

### Inclusion and exclusion criteria

We included randomized, quasi-randomized, or controlled clinical trials published in English and conducted in economically developed countries identified as such by the International Monetary Fund or the Organization for Economic Cooperation and Development [27]. We excluded non-English articles, with the rationale that non-English studies comprise only 1% of the EPOC registry. Because our goal was to understand interventions in routine outpatient practice, and to have uniformity in the types of settings included, we excluded studies conducted in nursing home or palliative care settings. To focus on the impact of interventions of process of care or patient outcomes, we excluded studies reporting only the following non-clinical outcomes: patient or provider knowledge; self-efficacy; satisfaction; or other attitudes and beliefs. To minimize heterogeneity among study populations, we excluded studies of *cor pulmonale *patients exclusively. Finally, to focus on interventions that attempted to improve care by changing the organizations or settings in which care was delivered, we excluded: care pathway interventions without organizational components (*e.g*., patient or provider education only); work site health interventions; exercise rehabilitation or diet only; and disease prevention or screening only.

Four investigators independently reviewed overlapping groups of differing halves of the citations' titles and abstracts generated by the full literature search to assess agreement regarding potentially eligible publications. If eligibility was uncertain after review of the title and abstract, the full article was reviewed. Eligible studies were independently reviewed and jointly abstracted in detail by teams of two investigators. Disagreements were resolved by consensus of the group of investigators.

### Assessment of leveraging of characteristics of CAS

Eligible publications of organizational interventions as defined by the inclusion and exclusion criteria were then independently evaluated by two raters with content expertise in complexity science to assess the extent to which each reported intervention utilized the following four recognized CAS characteristics [15,19,22]: individuals' capacity/ability to learn; the interconnections between individuals; the ability of participants to self-organize; and the tendency of participants to co-evolve. Each intervention was given a point for each of the characteristics present in the study design, for a possible lowest score of 0 and highest score of 4. If a study contained more than one intervention, each was assessed separately. Definitions of each characteristic, along with examples of specific interventions felt to reflect each characteristic, are summarized in Table 1. An example of an intervention felt to include all four CAS characteristics included the addition of a nurse practitioner-led clinic (changing the interconnections between patients and providers), protocol development, and patient education (learning). Patients received individualized feedback (self-organization), and the frequency of visits and type of feedback changed depending on the patient's progress or symptoms (self-organization and co-evolution). An intervention that included only one CAS element was one in which data from a one-time patient survey was used to generate standardized care suggestions embedded within an electronic health record (only assessed as changing interconnections among patients and providers by adding a new contact point). Additional File 2 contains detail of each reported intervention and its CAS characteristic rating. The raters were blinded to the outcomes of the studies. The kappa for these scores between reviewers was 0.84, with conflicts subsequently resolved by discussion.

**Table 1:** Characteristics of Complex Adaptive Systems Abstracted

Characteristic	Definition	Example
Agents who Learn	• People can and will process information, as well as react to changes in information	• 'Health Buddy' with educational content • Teach guidelines

Interconnections	• Change in pattern of interactions, including non-verbal communication, among agents • Introducing new agents into the system	• Letters to patients • Nurse-led heart failure group clinic • Clinical reminders
Self-organization	• Order is created in a system without explicit hierarchical direction	• Flexibility in tailoring intervention to individual patients
Co-evolution	• The system and the environment influence each other's development	• Individualized 'HOME' treatment plan that changes over time

### Assessment of reported outcomes

Because of the great heterogeneity among reported outcomes, we did not use effect size as the outcome variable. Instead, we used a rating scale to assess the effectiveness of the intervention. The outcomes of each study were rated by two independent reviewers on a scale of 0 (no effect), 0.5 (mixed results), and 1 (intervention effective) based on the type (process versus outcome), number, and statistical significance of outcomes reported. Table 2 summarizes the criteria for each rating category, as well as provides examples of outcomes felt to reflect each category of effectiveness. Reviewers were blinded to study intervention, author, and title of manuscript, and one outcome rater was different from the intervention raters. The kappa for these scores was 0.86 with conflicts resolved by discussion.

**Table 2:** Criteria used to classify intervention effectiveness, with examples of outcomes reflecting each level of effectiveness

Outcome Score	Criteria	Example
0	• No statistically differences between control and intervention groups, or between intervention and baseline, on process or outcome measures	• No difference in adherence, NYHA class, # visits, or # hospitalizations

0.5	• Trends without significance • Mixed outcomes (significant improvement in minority of measures) • Significant improvement compared with baseline, but not with control	• Significant improvement in adherence, trends for CHF-related admission and total number of hospital days

1	• Statistically significant improvement: -all outcomes if ≤3 endpoints -majority of outcomes if > 3 endpoints	• Significant reduction in all-cause mortality and all-cause admissions at 3 months

### Statistical analysis of association between CAS characteristics and intervention effectiveness

We used Fisher's exact test to test the significance of the relationship between total number of characteristics of CASs utilized in an intervention and the strength of outcomes reported, as well as between each individual characteristic and the strength of outcomes. Because a mismatch between the unit of allocation and analysis may bias a study towards positive results, we divided studies into two groups based on whether a unit of analysis error was present. A second analysis using Fisher's exact test was performed including only those studies that did not contain a unit of analysis error. Finally, a third analysis using logistic regression was performed to weight studies based on both sample size and duration of intervention, with outcome rating as the independent variable, and CAS score, sample size, and intervention duration as independent predictors.

All statistical analysis was performed using Stata 9.2 (College Station, Texas).

### Analysis contrasting CAS characteristics associated with effectiveness for CHF and diabetes

The relationship between each individual CAS characteristic utilized in an intervention and strength of reported outcomes was assessed, using Fisher's exact test with and without intervention with unit of analysis error, as well as logistic regression. The individual characteristics associated with intervention effectiveness for CHF were compared with those previously reported for diabetes [22].

## Results

### Systematic review of organizational interventions for CHF

**Figure 1:**
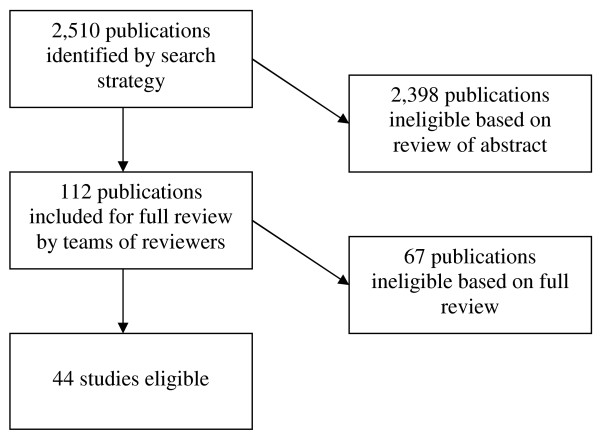
**Articles eligible and ineligible at each stage of review**.

Our search identified 2,510 publications for CHF. Of those, 112 were potentially eligible based on review of title and abstract, and were fully reviewed by a team of two investigators. Based on this review, 44 articles were eligible for inclusion [28-71]. Figure 1 details the numbers of articles eligible and ineligible at each stage of review. Seven studies had unit of allocation error, in which the unit of randomization was either the provider or clinic, but the unit of analysis was the patient. Two studies reported two distinct interventions [50,59]; each intervention was analyzed separately for a total of 46 interventions across 44 studies. The CAS scores for each study are contained in Additional File 2.

Only 13 interventions out of 46 (28%) received a rating of 1 for outcomes through demonstrating significant improvement in most or all reported outcomes; all others were felt to have mixed or negative results. All interventions incorporated at least one CAS characteristic, with 41% utilizing two CAS characteristics, 28% utilizing three, and 24% utilizing four. Ninety-three percent of reported interventions were judged to change the pattern of interconnections between individuals, typically through the introduction of a new person such as a case manager. Thirty-five interventions (76%) impacted learning; sixteen (35%) allowed self-organization of study participants; and in thirty (65%), the intervention evolved over time based on factors such as the patient's status or symptoms.

The association between number of CAS characteristics leveraged in an intervention and its effectiveness is shown in Table 3. None of the studies utilizing only one or two characteristics demonstrated significant improvement in most or all outcomes. All studies utilizing three or four CAS characteristics demonstrated at least mixed results, and ninety-one percent of those using all four CAS characteristics received the highest rating of effectiveness based on having demonstrated statistically significant improvement in most or all outcomes. This association between number of CAS characteristics utilized and the effectiveness of an intervention was significant (p < 0.001), and remained so after studies with unit of allocation error were excluded (p < 0.001). This association also remained significant in logistic regression analysis, adjusting for sample size and intervention duration (p < 0.001).

**Table 3:** Distribution of CAS and intervention effectiveness for CHF studies

Total CAS Score	Rating of Intervention Effectiveness			Total No. Studies with each CAS Score
	**0**	**0.5**	**1**	
**1**	1	2	0	3
**2**	6	13	0	19
**3**	0	10	3	13
**4**	0	1	10	11
**Total**	**7**	**26**	**13**	**46**

P < 0.001

Three individual CAS characteristics were associated with CHF intervention effectiveness: learning (p = 0.05), self-organization (p < 0.001), and co-evolution (p = 0.002). These associations remained significant after excluding studies with unit of analysis error. The association between interconnections and effectiveness was not significant (p = 0.72). The detail of analysis for individual CAS characteristics and intervention effectiveness is shown in Additional File 3.

### Analysis contrasting CHF and diabetes

A systematic review of interventions to improve care of patients with diabetes through changing the way health care organizations delivered care was previously published. The methods for that review were identical to those reported here with the exception of the disease-specific search terms used and 32 studies were identified. In that review, the presence of CAS characteristics and effectiveness of interventions were also assessed, and the association between the two was performed with Fisher's exact test, with and without studies with unit of allocation error. We used those data to compare the differences between the individual CAS characteristics associated with intervention effectiveness for CHF and diabetes.

The association of individual CAS characteristics with intervention effectiveness between studies of organizational interventions for patients with type 2 diabetes, and those with CHF is shown in Table 4. Only the CAS characteristic 'co-evolution' was significantly associated with intervention effectiveness in both diabetes and CHF. Learning and self-organization were associated intervention effectiveness for CHF, and interconnections were associated with intervention effectiveness for diabetes.

**Table 4:** Association between individual CAS characteristic and intervention effectiveness for type 2 diabetes [22] and CHF

CAS characteristic	type 2 diabetes^22^		CHF	
	
	Proportion of studies utilizing	Association with effectiveness	Proportion of studies utilizing	Association with effectiveness
Learning	80%	p = 0.07	76%	p = 0.05

Interconnections	77%	p = 0.05	93%	P = 0.72

Self-organization	27%	p = 0.58	35%	p < 0.001

Co-evolution	70%	p = 0.003	65%	p = 0.002

## Discussion

This systematic review of interventions to improve outcomes of patients with CHF through changing care delivery processes in clinical settings is consistent with literature reporting that interventions that attempt to improve patient outcomes through impacting the organizations in which care is delivered have mixed results [2,4-6]. In this review, the majority (72%) of interventions were not effective in significantly improving outcomes. Our analysis of these interventions through the lens of a CAS perspective again demonstrates that interventions consistent with a CAS perspective are more likely to be effective in improving outcomes. We interpret this as providing further evidence that the clinical settings are CASs. For interventions to be effective in improving patient outcomes, they must take this into account.

The difference in the individual CAS characteristics associated with intervention effectiveness for patients with type 2 diabetes and CHF brings a new perspective to the consideration of clinical settings as CASs. Specifically, we believe that when implementing interventions to improve outcomes of patients with chronic disease, not only must the characteristics of the organization be considered, but so must the characteristics of the disease and its treatment. Interventions must be appropriately matched to the level of complexity of not only the organization, but also of the disease, as disease and treatment characteristics may influence what interventions are more likely to be effective.

The interplay of differences between chronic diseases within the context of CAS clinical systems will affect which approaches are more or less likely to be effective for patients with a specific disease. The level of uncertainty inherent in diseases and their treatments may be an important contributor to these differences. To illustrate this point, Table 5 outlines how potential differences between type 2 diabetes and CHF in terms of uncertainty may influence the CAS characteristics that were associated with intervention effectiveness for each disease. For example, the myriad combinations of lifestyle and medication approaches to managing type 2 diabetes may be more complex and nuanced than those for CHF, and the symptoms of worsening glycemic control may be more subtle and insidious than worsening volume status, leading to greater uncertainty in the management of diabetes relative to CHF [72]. This greater uncertainty may influence the effectiveness of CAS characteristics in interventions, or the effectiveness of combinations of characteristics. Because one way that individuals can navigate uncertainty is through relationships, interconnections may be particularly important with increasing uncertainty.

**Table 5:** Potential differences between type 2 diabetes and CHF with regards to uncertainty, and how they might influence CAS characteristic effectiveness

CAS characteristic	type 2 diabetes	CHF
Learning	Treatment is nuanced and complex, making efforts to improve outcomes through learning more difficult.	Less uncertainty in treatment guidelines allows more prescriptive, algorithmic approaches to management that may be more easily learned.

Interconnections	Greater degree of uncertainty in terms of symptoms and management, leading to greater reliance on interconnections to manage disease.	Lesser degree of uncertainty in terms of symptoms and management may lead interventions focused on interconnections less effective.

Self-organization	Greater uncertainty in management and symptoms of exacerbation may make efforts to self-organize more difficult.	Less uncertainty regarding management and symptoms of exacerbation may make efforts to self-organize more effective.

Co-evolution	Course and symptoms evolve over time in unique trajectory.	Course and symptoms evolve over time in unique trajectory.

However, for both CHF and type 2 diabetes, patients have a chronic disease that is changing over time, and the recognition of the dynamic nature of the evolution of disease in interventions is important. Also in both cases, the fact that the clinical settings in which patients receive care are CASs is an important contextual consideration, as no two are exactly alike.

The implication of these findings for implementation research whose goal is to change organizations to improve care of patients with chronic disease is that we must shift our focus in intervention design. While considerations such as cost, ease of implementation, and level of disruption to the clinical setting are important, the levels of complexity of the organization and the disease are even more important. Intervention design for chronic disease requires a greater level of nuance, individualization, flexibility, and assessment over time. Specific implications of this insight include the need to pay attention to or explicitly change the relationships between individuals as a strategy to improve outcomes, the importance of allowing 'local' input or control into the intervention design, and the need to provide feedback regarding the impact of the intervention and the possibility to change the intervention based on this feedback.

This study has several limitations. The first is the relatively small number of studies of organizational interventions. However, despite these small numbers, the associations found are significant ones. A more overarching limitation is the difficulty in applying the lens of a CASs perspective to traditional intervention design, with specific regard to assigning scores regarding CAS characteristics retrospectively. Our method of independent review of interventions and results using groups of separate reviewers was intended to offset this methodological limitation, and our kappa scores suggest that reviewers did have a consistent ability to make these retrospective assessments.

Other limitations include the possibility of publication bias, which may have led either to negative studies not being published, or to the interventions being described in less detail, making assessment of CAS characteristics more difficult. Negative studies are well represented in the distribution of outcomes in the included studies, and the inter-rater consistency suggests that sufficient information was available to make an assessment. All raters are from the same institution, and the possibility of bias in terms of the CAS characteristics on which we focused is possible, but made less likely by our use of characteristics consistently recognized as key in the CAS literature.

Finally, this analysis is limited to studies of patients with type 2 diabetes and CHF. The applicability of our findings to other chronic diseases, or to acute disease processes, has yet to be demonstrated. However, we believe that the strength of our results across a combined 76 examples of interventions (46 CHF and 32 diabetes) for two distinct chronic diseases is at least suggestive of the range of application of the CAS framework in clinical settings.

## Summary

The significant association between CAS characteristics and effectiveness of reported outcomes for patients with CHF builds on the idea that for interventions to be effective, they must be consistent with the CAS nature of the clinical systems in which they are applied. However, the fact that different CAS characteristics are associated with intervention effectiveness for CHF and type 2 diabetes suggests that the nature of the diseases being treated may mediate the intervention effectiveness. We suggest that the level of uncertainty associated with a disease and its treatment may be an important characteristic that must be considered in designing interventions to lead to the greatest improvement in patient outcomes.

## Competing interests

The authors declare that they have no competing interests.

## Authors' contributions

LKL conceived this analysis using the database conceived by VL, PN, and JP, rated studies, performed preliminary statistical analysis, interpreted findings, and drafted the manuscript. MP rated studies, interpreted findings, and helped to draft the manuscript. JP participated in the design of the study and helped to draft the manuscript. VL conceived the systematic review and database, rated studies, and helped to draft the manuscript. PN conceived the systematic review and database and helped to draft the manuscript. RMcD participated in the design of the study, provided theoretical expertise, interpreted findings, and helped to draft the manuscript. All authors have read and approved the final manuscript.

## Supplementary Material

Additional file 1 **Search strategy to identify studies of organizational interventions to improve outcomes for patients with congestive heart failure**. Search completed 17 July 2008. Additional file 1 is a word document detailing the keywords and number of results identified by each keyword used in our search strategy. Click here for file

Additional file 2 **Summary of eligible studies of organizational interventions on outcomes of patients with congestive heart failure**. Additional file 2 is a word document listing each eligible study, along with details regarding sample size, our ratings of its intervention and the number of CAS characteristics leveraged, follow-up duration, and presence or absence of unit of analysis error. Click here for file

Additional file 3 **Detail of analysis for individual CAS characteristics and intervention effectiveness for CHF**. Additional file 3 is a table in word document format that lists each CAS characteristic, the number of studies in which the characteristic was utilized, and the range of intervention effectiveness scores for those studies. Click here for file
