# Supplementary material for: The importance of organizational characteristics for improving outcomes in patients with chronic disease: a systematic review of congestive heart failure
Source: Implement Sci. 2010 Aug 25;5:66. doi: 10.1186/1748-5908-5-66 (PMC2936445; doi:10.1186/1748-5908-5-66)
Supplement: Additional file 1 — Search strategy to identify studies of organizational interventions to improve outcomes for patients with congestive heart failure. Search completed 17 July 2008. Additional file 1 is a word document detailing the keywords and number of results identified by each keyword used in our search strategy. [file 1748-5908-5-66-S1.DOC]

**Additional File 1. Search strategy to identify studies of organizational interventions to improve outcomes for patients with congestive heart failure. Search completed 17** July 2008.

| Search History | | |
| --- | --- | --- |
| Line # | Key Words | Results |
| 1 | Quality of Health Care/ | 45,354 |
| 2 | Quality Assurance, Health Care/ | 40,754 |
| 3 | Quality Indicators, Health Care/ | 6,441 |
| 4 | Quality Control/ | 33,801 |
| 5 | (continuous adj5 quality adj improvement).tw. | 1,696 |
| 6 | (institute adj2 healthcare adj improvement$).tw. | 184 |
| 7 | (total adj quality adj improvement$).tw. | 42 |
| 8 | (practice adj2 design$).tw. | 1,433 |
| 9 | (practice adj2 redesign$).tw. | 46 |
| 10 | (practice adj2 engineer$).tw. | 83 |
| 11 | (practice adj2 reengineer$).tw. | 7 |
| 12 | (professional adj substitution$).tw. | 4 |
| 13 | (boundary adj encroachment).tw. | 2 |
| 14 | Program Evaluation/ | 34,730 |
| 15 | PDSA.tw. | 71 |
| 16 | Practice Guidelines/ | 55,477 |
| 17 | (practice adj guideline$).pt,tw. | 8,918 |
| 18 | Guideline Adherence/ | 12,651 |
| 19 | (guideline$ adj5 implement$).tw. | 4,158 |
| 20 | (group adj visit$).tw. | 149 |
| 21 | Case Management/ | 6,828 |
| 22 | Decision Making, Organizational/ | 9,588 |
| 23 | Organizational Innovation/ | 17,589 |
| 24 | Organizational Case Studies/ | 6,639 |
| 25 | Organizational Culture/ | 9,605 |
| 26 | models, organizational/ | 11,590 |
| 27 | (organizational adj development$).tw. | 247 |
| 28 | (organizational adj change$).tw. | 1,109 |
| 29 | (organizational adj improvement$).tw. | 62 |
| 30 | (organizational adj intervention$).tw. | 91 |
| 31 | (organizational adj learning).tw. | 118 |
| 32 | (organizational adj2 objective$).tw. | 65 |
| 33 | (organizational adj2 outcome$).tw. | 167 |
| 34 | (organizational adj2 transformation$).tw. | 47 |
| 35 | organizational$.tw. | 20,601 |
| 36 | "Outcome Assessment (Health Care)"/og [Organization & Administration] | 1,256 |
| 37 | "Process Assessment (Health Care)"/og [Organization & Administration] | 186 |
| 38 | Outpatient Clinics, Hospital/og [Organization & Administration] | 2,049 |
| 39 | Registries/ or registr$.tw. | 82,812 |
| 40 | Clinical Pathways/ | 3,359 |
| 41 | (shared adj care).tw. | 598 |
| 42 | or/1-41 | 360,037 |
| 43 | Disease Management/ | 6,934 |
| 44 | Patient Care Planning/ | 29,734 |
| 45 | Patient-Centered Care/ | 6,091 |
| 46 | Primary Health Care/ | 41,982 |
| 47 | Progressive Patient Care/ | 1,129 |
| 48 | Critical Pathways/ | 3,359 |
| 49 | Delivery of Health Care, Integrated/ | 6,045 |
| 50 | Health Services Accessibility/ | 37,044 |
| 51 | Managed Care Programs/ | 22,675 |
| 52 | Product Line Management/ | 1,474 |
| 53 | Patient Care Team/ | 43,345 |
| 54 | Behavior Control/ | 1,109 |
| 55 | Counseling/ | 23,541 |
| 56 | Health Promotion/ | 38,192 |
| 57 | Patient Compliance/ | 39,031 |
| 58 | After-Hours Care/ | 541 |
| 59 | (coordination or coordinated or multifactorial or multi-factorial or multicomponent or multi-component or multidisciplinary or multi-disciplinary or interdisciplinary or inter-disciplinary or integrated or community-based or organized).tw. | 255,398 |
| 60 | (care or approach or intervention or strategy or strategies or management or managing or center$ or clinic).tw. | 2,079,734 |
| 61 | 59 and 60 | 100,046 |
| 62 | "Organization and Administration".sh. | 14,365 |
| 63 | or/43-58,61-62 | 368,466 |
| 64 | Total Quality Management/ | 10,880 |
| 65 | Quality Control/ | 33,801 |
| 66 | (tqm or tqi or cqu).tw. | 604 |
| 67 | (quality and (continuous or total)).tw. | 50,945 |
| 68 | (total and (management or improvement)).tw. | 69,465 |
| 69 | or/64-68 | 151,900 |
| 70 | Education, Continuing/ | 6,712 |
| 71 | (education and continuing and (medical or professional$ or nursing or physician$ or nurse$)).tw. | 8,829 |
| 72 | (outreach and (visit or educational)).tw. | 761 |
| 73 | (academic and detailing).tw. | 271 |
| 74 | or/70-73 | 16,005 |
| 75 | Diffusion of Innovation/ | 9,941 |
| 76 | (diffusion and (innovation or technology)).tw. | 1,362 |
| 77 | or/75-76 | 10,902 |
| 78 | Medical Audit/ | 12,797 |
| 79 | (audit or feedback or compliance or adherence or training).tw. | 329,646 |
| 80 | (improvement$ or improving or improves or improve or guideline$ or practice$ or medical or provider$ or physician$ or nurse$ or clinician$).tw. | 1,790,439 |
| 81 | 79 and 80 | 129,688 |
| 82 | Practice Guidelines/ | 55,477 |
| 83 | (academic or visit$ or reminder$).tw. | 171,344 |
| 84 | Reminder Systems/ | 1,525 |
| 85 | ((financial or economic or physician$ or patient$) and incentive$).tw. | 5,973 |
| 86 | Reimbursement Mechanisms/ | 8,891 |
| 87 | or/78,81-86 | 360,182 |
| 88 | Medical Informatics/ | 5,353 |
| 89 | (computer or (decision and support)).tw. | 136,833 |
| 90 | Telemedicine/ | 7,369 |
| 91 | (telemedicine or telecommunication$ or web or modem or telephone$).tw. | 60,763 |
| 92 | Internet/ | 33,078 |
| 93 | Telephone/ | 7,595 |
| 94 | or/88-93 | 222,782 |
| 95 | or/63,69,74,77,81,87,94 | 1,004,803 |
| 96 | outreach.tw. | 5,343 |
| 97 | ((opinion or education$ or influential) adj leader$).tw. | 692 |
| 98 | facilitator$.tw. | 8,484 |
| 99 | (academic adj detailing).tw. | 223 |
| 100 | (consensus adj conference).tw. | 2,866 |
| 101 | *Guideline Adherence/ | 5,933 |
| 102 | Practice Guidelines/ | 55,477 |
| 103 | (practice adj guideline$).tw. | 8,918 |
| 104 | (guideline$ adj2 (introduc$ or issu$ or impact or effect$ or disseminat$ or distribut$)).tw. | 2,714 |
| 105 | ((effect$ or impact or evaluat$ or introduc$ or compar$) adj2 training program$).tw. | 559 |
| 106 | *Reminder Systems/ | 880 |
| 107 | reminder$.tw. | 4,602 |
| 108 | (recall adj2 system$).tw. | 296 |
| 109 | (prompter$ or prompting).tw. | 3,038 |
| 110 | algorithm$.tw. | 72,928 |
| 111 | or/96-110 | 161,688 |
| 112 | *Feedback/ or feedback.tw. | 55,896 |
| 113 | (feedback adj (loop$ or control$ or regula$ or mechanism$ or inhib$ or system$ or circuit$ or sensory or visua or audio$ or auditory)).tw. | 20,754 |
| 114 | 112 not 113 | 35,142 |
| 115 | (chart adj review$).tw. | 14,264 |
| 116 | ((effect$ or impact or records or chart$) adj2 audit).tw. | 696 |
| 117 | compliance.tw. | 60,185 |
| 118 | marketing.tw. | 12,498 |
| 119 | or/115-118 | 87,048 |
| 120 | exp *Reimbursement Mechanisms/ | 14,248 |
| 121 | (fee adj2 service).tw. | 2,683 |
| 122 | *Capitation Fee/ | 1,970 |
| 123 | "Deductibles and Coinsurance"/ | 1,247 |
| 124 | (cost adj shar$).tw. | 843 |
| 125 | (copayment$ or (co adj payment$)).tw. | 828 |
| 126 | (prepay$ or prepaid or (prospective adj payment$)).tw. | 3,845 |
| 127 | *Hospital Charges/ | 726 |
| 128 | formular$.tw. | 2,714 |
| 129 | fundhold$.tw. | 375 |
| 130 | *Medicaid/ | 8,548 |
| 131 | *Medicare/ | 15,088 |
| 132 | Blue Cross/ | 2,248 |
| 133 | or/120-132 | 44,777 |
| 134 | *Nurse Clinicians/ | 5,037 |
| 135 | *Nurse Practitioners/ | 9,084 |
| 136 | *Nurses' Aides/ | 2,149 |
| 137 | (nurse adj (rehabilitator$ or clinician$ or practitioner$)).tw. | 6,600 |
| 138 | *Pharmacists/ | 5,169 |
| 139 | (clinical adj pharmacist$).tw. | 853 |
| 140 | paramedic$.tw. | 4,462 |
| 141 | psychologist$.tw. | 7,606 |
| 142 | social workers.tw. | 3,883 |
| 143 | dietician$.tw. | 631 |
| 144 | *Physician Assistants/ | 2,401 |
| 145 | *Patient Care Team/ | 16,780 |
| 146 | (team$ adj2 (care or treatment)).tw. | 6,273 |
| 147 | (integrat$ adj (care or service$)).tw. | 1,617 |
| 148 | (care adj (coordinat$ or program$ or continuity)).tw. | 7,218 |
| 149 | *Case Management/ | 4,208 |
| 150 | exp *Ambulatory Care Facilities/ | 21,488 |
| 151 | *Ambulatory Care/ | 13,369 |
| 152 | or/134-151 | 106,079 |
| 153 | *Home Care Services/ | 17,120 |
| 154 | *Nursing Homes/ | 16,857 |
| 155 | *Office Visits/ | 1,762 |
| 156 | *House Calls/ | 1,082 |
| 157 | *Day Care/ | 2,696 |
| 158 | *Aftercare/ | 2,421 |
| 159 | *Community Health Nursing/ | 13,508 |
| 160 | (chang$ adj1 location$).tw. | 236 |
| 161 | domiciliary.tw. | 1,942 |
| 162 | (home adj treat$).tw. | 1,083 |
| 163 | (day adj surgery).tw. | 1,630 |
| 164 | *Medical Records/ | 14,948 |
| 165 | *Medical Records, Computerized/ | 11,370 |
| 166 | (information adj2 (management or system$)).tw. | 18,834 |
| 167 | *Peer Review/ | 2,790 |
| 168 | *Utilization Review/ | 2,445 |
| 169 | *Health Services Misuse/ | 1,688 |
| 170 | or/153-169 | 105,520 |
| 171 | *Physician's Practice Patterns/ | 17,491 |
| 172 | *Quality Assurance, Health Care/ | 23,036 |
| 173 | *"Process Assessment (Health Care)"/ | 1,094 |
| 174 | *ProGram Evaluation/ | 5,292 |
| 175 | *Length of Stay/ | 5,523 |
| 176 | (early adj discharge).tw. | 1,523 |
| 177 | (discharge adj planning).tw. | 1,717 |
| 178 | offset.tw. | 12,612 |
| 179 | triage.tw. | 6,066 |
| 180 | exp *"Referral and Consultation"/ | 18,592 |
| 181 | *Drug Therapy, Computer Assisted/ | 896 |
| 182 | (near adj patient adj testing).tw. | 156 |
| 183 | *Medical History Taking/ | 3,836 |
| 184 | *Telephone/ | 3,548 |
| 185 | *Physician-Patient Relations/ | 23,835 |
| 186 | *Health Maintenance Organizations/ | 9,306 |
| 187 | *Managed Care Programs/ | 15,522 |
| 188 | (hospital$ adj merg$).tw. | 290 |
| 189 | or/171-188 | 144,153 |
| 190 | ((standard or usual or routine or regular or traditional or conventional or pattern) adj care).tw. | 8,393 |
| 191 | (program$ adj (reduc$ or increas$ or decreas$ or chang$ or improv$ or modify$ or monitor$ or care)).tw. | 3,471 |
| 192 | (program adj (health or care or intervention)).tw. | 335 |
| 193 | (((effect$ or impact or evauat$ or introduc$ or compar$) adj (treatment or care or screening or prevent$)) and program$).tw. | 2,704 |
| 194 | (computer$ adj (dosage or dosing or diagnosis or therapy or decision$)).tw. | 671 |
| 195 | (protocol$ adj (introduc$ or impact or effect$ or implement$ or computers$)).tw. | 321 |
| 196 | ((effect$ or impact or introduc$) adj (legislation or regulation$ or policy)).tw. | 623 |
| 197 | Community Health Services/ | 23,739 |
| 198 | Research/og | 2,328 |
| 199 | "Outcome and Process Assessment (Health Care)"/ | 18,079 |
| 200 | or/190-199 | 59,796 |
| 201 | or/111,114,119,133,152,170,189,200 | 665,985 |
| 202 | or/42,95,201 | 1,531,278 |
| 203 | exp Heart Failure, Congestive/ | 68,638 |
| 204 | (congestive adj heart failure).tw. | 26,868 |
| 205 | (heart adj failure).tw. | 76,158 |
| 206 | CHF.tw. | 8,140 |
| 207 | exp Myocardial Diseases/ | 61,569 |
| 208 | Cardiomyopathy, Hypertrophic, Familial/ | 310 |
| 209 | Cardiomyopathy, Restrictive/ | 589 |
| 210 | Chagas Cardiomyopathy/ | 1,443 |
| 211 | Endocardial Fibroelastosis/ | 958 |
| 212 | Endomyocardial Fibrosis/ | 1,611 |
| 213 | Kearns Syndrome/ | 559 |
| 214 | Myocardial Reperfusion Injury/ | 8,929 |
| 215 | Myocarditis/ | 10,080 |
| 216 | or/208-215 | 24,028 |
| 217 | 207 not 216 | 37,541 |
| 218 | Cardiac Output, Low/ | 5,202 |
| 219 | exp Cardiomegaly/ | 39,950 |
| 220 | exp Ventricular Dysfunction/ | 18,660 |
| 221 | or/203-206,217-220 | 169,987 |
| 222 | 202 and 221 | 14,955 |
| 223 | limit 222 to yr=1989-2008 | 12,731 |
| 224 | limit 223 to english language | 11,059 |
| 225 | limit 224 to human | 10,388 |
| 226 | 225 not (child or infant$ or neonat$ or adolesc$ or pediatr$).sh,pt,tw,jn. | 9,482 |
| 227 | 226 not (letter or editorial or comment).sh,pt,tw. | 8,020 |
| 228 | 227 not Case Report.sh,pt,tw. | 7,993 |
| 229 | Randomized Controlled Trials/ | 67,227 |
| 230 | Randomized Controlled Trial.pt. | 294,484 |
| 231 | exp Controlled Clinical Trials/ | 0 |
| 232 | Controlled Clinical Trial.pt. | 83,667 |
| 233 | Random Allocation/ | 68,215 |
| 234 | Double-Blind Method/ | 108,500 |
| 235 | Single-Blind Method/ | 14,086 |
| 236 | or/229-235 | 495,771 |
| 237 | 228 and 236 | 1,106 |
| 238 | exp Clinical Trials/ | 0 |
| 239 | Clinical Trial.pt. | 475,065 |
| 240 | (clin$ adj trial$).tw. | 142,357 |
| 241 | ((singl$ or doubl$ or trebl$ or tripl$) adj25 (blind$ or mask)).tw. | 105,835 |
| 242 | Placebos/ | 29,705 |
| 243 | placebo$.tw. | 125,797 |
| 244 | random$.tw. | 483,725 |
| 245 | exp Research Design/ | 271,168 |
| 246 | or/238-245 | 1,024,699 |
| 247 | 228 and 246 | 2,097 |
| 248 | Comparative Study/ | 1,506,000 |
| 249 | exp Evaluation Studies/ | 133,825 |
| 250 | Follow-Up Studies/ | 407,658 |
| 251 | Prospective Studies/ | 280,867 |
| 252 | Multicenter Studies/ | 120,350 |
| 253 | (control$ or prospectiv$ or volunteer$).tw. | 2,214,915 |
| 254 | or/248-253 | 3,832,187 |
| 255 | 228 and 254 | 3,725 |
| 256 | (time adj series).tw. | 9,176 |
| 257 | ((pre adj test) or pretest or (post adj test) or posttest).tw. | 10,344 |
| 258 | (case adj (study or studies)).tw. | 33,889 |
| 259 | or/256-258 | 53,179 |
| 260 | 228 and 259 | 85 |
| 261 | or/237,247 | 2,227 |
| 262 | or/255,260 | 3,781 |
| 263 | 261 and 200901$.em. | 28 |
| 264 | 261 and 200902$.em. | 19 |
| 265 | 261 and 200903$.em. | 16 |
| 266 | 261 and 200904$.em. | 2 |
| 267 | 261 and 200905$.em. | 2 |
| 268 | 261 and 200806$.em. | 11 |
| 269 | 261 and 200807$.em. | 27 |
| 270 | 261 and 200808$.em. | 12 |
| 271 | 261 and 200809$.em. | 22 |
|  |  |  |
