# Supplementary material for: The importance of organizational characteristics for improving outcomes in patients with chronic disease: a systematic review of congestive heart failure
Source: Implement Sci. 2010 Aug 25;5:66. doi: 10.1186/1748-5908-5-66 (PMC2936445; doi:10.1186/1748-5908-5-66)
Supplement: Additional file 2 — Summary of eligible studies of organizational interventions on outcomes of patients with congestive heart failure. Additional file 2 is a word document listing each eligible study, along with details regarding sample size, our ratings of its intervention and the number of CAS characteristics leveraged, follow-up duration, and presence or absence of unit of analysis error. [file 1748-5908-5-66-S2.DOCX]

**Additional File 2. Summary of eligible studies of organizational interventions on outcomes of patients with congestive heart failure**

| **Author** | **Sample size** | **Intervention** | **CAS leveraged** | **Follow-up duration** | **Unit of Analysis error** |
| --- | --- | --- | --- | --- | --- |
| Rich^28^ | 282 | L,IC,SO,CO-E | 4 | 3 mo | N |
| Ekman^29^ | 158 | IC,CO-E | 2 | 6 mo | N |
| Cline^30^ | 190 | L,IC,CO-E | 3 | 1 yr | N |
| Jaarsma^31^ | 179 | L,IC | 2 | 9 mo | N |
| Stewart^32^ | 97 | IC, Co-E | 2 | 18 mos | N |
| Gattis^33^ | 181 | L,IC,CO-E | 3 | 6 mo | N |
| Stewart^34^ | 200 | L,IC,SO,Co-E | 4 | 12 mos | N |
| De Lusingnan^35^ | 20 | IC | 1 | 1 yr | N |
| Pugh^36^ | 58 | L,IC | 2 | 6 mo | N |
| Jerant^37^ | 37 | L,IC | 2 | 6 mo | N |
| Blue^38^ | 165 | L,IC | 2 | 1 yr | N |
| McDonald^39^ | 98 | L, IC | 2 | 3 mos | N |
| Doughty^40^ | 197 | L,IC,CO-E | 3 | 1 yr | Y |
| Kasper^41^ | 200 | IC,CO-E | 2 | 6 mo | N |
| Harrison^42^ | 200 | IC,CO-E | 2 | 6 wk | N |
| Capomolia^43^ | 234 | L,IC,SO,CO-E | 4 | 1 yr | N |
| Riegel^44^ | 228 | L,IC,SO,CO-E | 4 | 6 mo | N |
| Bouvy^45^ | 152 | L,IC | 2 | 6 mo | N |
| Bucci^46^ | 80 | IC,CO-E | 2 | 4 wk | N |
| Stromberg^47^ | 106 | L,IC,SO,CO-E | 4 | 1 yr | N |
| La Framboise^48^ | 90 | L,IC,CO-E | 3 | 8 wk | N |
| Barnason^49^ | 35 | L,IC | 2 | 3 mo | N |
| Ansari 1^50^ | 169 | L,IC | 2 | 1 yr | Y |
| Ansari 2^50^ | 169 | L,IC,SO,CO-E | 4 | 1 yr | Y |
| Goldberg^51^ | 280 | L,IC,CO-E | 3 | 6 mo | N |
| Laramee^52^ | 287 | L,IC,CO-E | 3 | 3 mo | N |
| Atienza^53^ | 338 | L,IC,SO,CO-E | 4 | 1 yr | N |
| Tsuyuki^54^ | 276 | L,IC | 2 | 6 mo | N |
| Feldman^55^ | 371 | L,IC,CO-E | 3 | 3 mo | Y |
| Kimmelstiel^56^ | 200 | L,IC,SO,CO-E | 4 | 1 yr | N |
| Subramanian^57^ | 715 | IC,CO-E | 2 | 1 yr | Y |
| Bruggink-Andre^58^ | 236 | L,IC,SO,CO-E | 4 | 1 yr | N |
| Murtaugh 1^59^ | 338 | L,IC | 2 | 6 wk | Y |
| Murtaugh 2^59^ | 338 | L,IC,SO | 3 | 6 wk | Y |
| Caldwell^60^ | 36 | L,IC | 2 | 3 mo | N |
| Nucifora^61^ | 163 | L,IC,CO-E | 3 | 6 mo | N |
| Inglis^62^ | 297 | L,IC,SO,Co-E | 4 | 6 mos | N |
| Spaeder^63^ | 49 | IC,CO-E | 2 | 3 mo | N |
| Pearson^64^ | 98 | L,IC,SO,CO-E | 4 | 7.5 yr | N |
| Triller^65^ | 154 | L,SO | 2 | 6 mo | N |
| Khunti^66^ | 364 | IC,SO,Co-E | 3 | 1 year | N |
| Rao^67^ | 113 | SO | 1 | 10 mo | N |
| Heidenreich^68^ | 1546 | L | 1 | 9 mo | N |
| Kashem^69^ | 48 | IC,SO,Co-E | 3 | 1 yr | N |
| Kwok^70^ | 105 | L,IC,CO-E | 3 | 6 mo | N |
| Azad^71^ | 91 | L,IC,CO-E | 3 | 6 mo | N |
