# Supplementary material for: The importance of organizational characteristics for improving outcomes in patients with chronic disease: a systematic review of congestive heart failure
Source: Implement Sci. 2010 Aug 25;5:66. doi: 10.1186/1748-5908-5-66 (PMC2936445; doi:10.1186/1748-5908-5-66)
Supplement: Additional file 3 — Detail of analysis for individual CAS characteristics and intervention effectiveness for CHF. Additional file 3 is a table in word document format that lists each CAS characteristic, the number of studies in which the characteristic was utilized, and the range of intervention effectiveness scores for those studies. [file 1748-5908-5-66-S3.DOCX]

**Additional File 3. Detail of analysis for individual CAS characteristics and intervention effectiveness for CHF**

| **CAS Characteristic in intervention** | **0** | **0.5** | **1** | **Total number of studies with each CAS characteristic score** |
| --- | --- | --- | --- | --- |
| **Learning present** | 4 | 6 | 1 | 11 |
| **Learning absent** | 3 | 20 | 12 | 35 |
|  |  |  |  |  |
| **Interconnections present** | 0 | 3 | 0 | 3 |
| **Interconnections absent** | 7 | 26 | 13 | 43 |
|  |  |  |  |  |
| **Self-organization present** | 7 | 21 | 2 | 30 |
| **Self-organization absent** | 0 | 5 | 11 | 16 |
|  |  |  |  |  |
| **Co-evolution present** | 4 | 12 | 0 | 16 |
| **Co-evolution absent** | 3 | 14 | 13 | 30 |
